# LUNCH—Lung Ultrasound for early detection of silent and apparent aspiratioN in infants and young CHildren with cerebral palsy and other developmental disabilities: study protocol of a randomized controlled trial

**DOI:** 10.1186/s12887-022-03413-z

**Published:** 2022-06-23

**Authors:** S Fiori, RT Scaramuzzo, E Moretti, C Amador, T Controzzi, A Martinelli, L Filippi, A Guzzetta, L Gargagni

**Affiliations:** 1 Department of Developmental Neuroscience, Istituto Di Ricovero E Cura a Carattere Scientifico (IRCCS) Fondazione Stella Maris, Pisa, Italy; 2 Department of Clinical and Experimental Medicine, University of Pisa, Pisa, Italy; 3 Department of Neonatal Intensive Care Unit, S. Chiara Hospital, Pisa, Italy; 4 Institute of Clinical Physiology, National Research Council of Italy (CNR), Pisa, Italy

**Keywords:** Dysphagia, Neurological impairment, Infant, Lung ultrasound (LUS), Aspiration, GERD

## Abstract

**Background:**

Children with neurological impairment may have dysphagia and/or gastro-esophageal reflux disease (GERD), which predispose to complications affecting the airways, increasing risk for aspiration-induced acute and chronic lung disease, or secondarily malnutrition, further neurodevelopmental disturbances, stressful interactions with their caregivers and chronic pain. Only multidisciplinary clinical feeding evaluation and empirical trials are applied to provide support to the management of feeding difficulties related to dysphagia or GERD, but no standardized feeding or behavioral measure exists at any age to assess aspiration risk and support the indication to perform a videofluoroscopic swallowing study (VFSS) or a fibre-optic endoscopic examination of swallowing (FEES), in particular in newborns and infants with neurological impairments. Lung ultrasound (LUS) has been proposed as a non-invasive, radiation-free tool for the diagnosis of pulmonary conditions in infants, with high sensitivity and specificity.

**Methods:**

A RCT will be conducted in infants aged between 0 and 6 years having, or being at risk for, cerebral palsy, or other neurodevelopmental disease that determines abnormal muscular tone or motor developmental delay assessed by a quantitative scale for infants or if there is the suspicion of GERD or dysphagia based on clinical symptoms. Infants will be allocated in one of 2 groups: 1) LUS-monitored management (LUS-m); 2) Standard care management (SC-m) and after baseline assessment (T0), both groups will undergo an experimental 6-months follow-up. In the first 3 months, infants will be evaluated a minimum of 1 time per month, in-hospital, for a total of 3 LUS-monitored meal evaluations. Primary and secondary endpoint measures will be collected at 3 and 6 months.

**Discussion:**

This paper describes the study protocol consisting of a RCT with two main objectives: (1) to evaluate the benefits of the use of LUS for monitoring silent and apparent aspiration in the management of dysphagia and its impact on pulmonary illness and growth and (2) to investigate the impact of the LUS management on blood sample and bone metabolism, pain and interaction with caregivers.

**Trial registration:**

Trial registration date 02/05/2020; ClinicalTrials.gov Identifier: NCT04253951.

## Background

Children with neurological impairment commonly experience abnormal ingestion functions (WHO, http://apps.who.int/classifications/icfbrowser/), including impairment in oral-motor function, rumination, abnormal swallowing, regurgitation and vomiting, defined as dysphagia and/or gastro-esophageal reflux disease (GERD), delayed gastric emptying and constipation [1, 2]. The number of these children is estimated not to decline in the next years, and their life expectancy to increase [3]. The neurological impairment may arise from a variety of disorders, including cerebral palsy or rare paediatric diseases such as genetic, metabolic, muscular and movement disorders. Primary or secondary muscle tone abnormalities in these disorders contribute to dysphagia and/or GERD, which predispose to complications affecting the airways, increasing risk for insidious development of aspiration-induced acute and chronic lung disease, or secondary malnutrition, further neurodevelopmental disturbances, and stressful interactions with their caregivers [4–6]. Moreover, infants with GERD likely experience chronic pain: inability to settle, grimacing, tense body, hypo- or hyper-reactions to acute pain, and deregulated sleep are possible indicators, even if no current definitions exist that are wholly applicable to infancy [7].

In such patients, a failure to thrive is the sum of nutritional and non-nutritional factors (mainly frequent respiratory infections and chronic pain, as well as use of antiepileptic medication). Malnutrition per se is due to feeding difficulties (with consequent insufficient dietary intake or excessive nutrient losses) and altered energy metabolism, and is associated to micronutrients deficiencies and osteopenia [8]. In the last couple of decades, the nutritional needs of children with neurological impairment have become a major matter of interest so that, in 2017, the European Society of Gastroenterology, Hepatology, and Nutrition (ESPGHAN) published a consensus statement (ESPGHAN guidelines) on the diagnosis and management of gastrointestinal and nutritional complications in children with neurological disability [9]. Both diagnosis of GERD and accurate monitoring of growth and nutritional status are among the “top ten-tips” in managing nutritional issues and gastrointestinal function in neurologically impaired children [10].

Oral fluid, food or refluxed gastric contents can be aspirated into the lungs, apparently, by choking or coughing, or silently. In order to directly or indirectly diagnose aspiration in dysphagia and GERD, video-fluoroscopic swallowing study (VFSS) and fiber-optic endoscopic examination of swallowing (FEES) are options, however they focus on a single study, thus clinical diagnosis is seldom made despite a normal result and, due to radiation exposure, invasiveness and costs, they are poorly reproducible in the same patient [11–13]. Furthermore, rheological and material property parameters among the barium-impregnated liquids and the thickened and unthicken infant formula are different, thus biasing the interpretation of the radiological results and consequent clinical recommendations [13, 14]. Finally, only clinical feeding evaluation and empirical trials are applied to provide support to the management of feeding difficulties related to dysphagia or GERD, and no standardized feeding or behavioral measure exists at any age to assess aspiration risk and support the indication to perform VFSS or FEES.

The picture is further complicated at young ages, as recently the concept of ‘early diagnosis’ has emerged for cerebral palsy and a variety of neurological disorders [15]. Indeed, most studies focusing on dysphagia and GERD in neurologically impaired children refer to ages older than 1 year. In the few studies investigating dysphagia in infants younger than 1 year by using VFSS, aspiration was present in 50% of infants with suspected dysphagia and up to 90% of them had silent aspiration [16]. This indicates that many infants who are not clinically suspected of having dysphagia or GERD may have silent aspiration, therefore a monitoring would be recommended in infants with higher aspiration risk, starting early from the first days of life. This would lead to earlier diagnosis, clinical management ad tailored habilitation and nutritional strategies to improve outcome and limit short-term and long-term complications [17].

Lung ultrasound (LUS) has been proposed and gained consensus as a non-invasive, radiation-free tool for the diagnosis of acute and chronic pulmonary conditions in both infants and adults, with high sensitivity and specificity in several conditions [18–22] LUS is an easy technique that may be performed during routine physical examination, with a point-of-care approach. Previous studies demonstrated that LUS is able to detect small lung consolidations with very high accuracy [22], and can be effectively used to follow-up the dynamic changes of these lesions. LUS is a patient-friendly tool, especially in young children, who are particularly susceptible to radiation exposure, which can impact later in their life with an increase risk of cancer [23].

The trial is conceived as a double-blinded randomized parallel-designed controlled trial (Consort checklist, [24]), comparing LUS-monitored management and standard care.

We hypothesize infants receiving LUS-monitored management having lower rate of pulmonary illness and better growth curves, and also reduce the need for invasive diagnostic (VFSS/FEES) over time. We also hypothesize infants receiving LUS-monitored management will have better biochemical and bone metabolism results, lower pain indices and less stressful interaction with caregivers.

## Methods

**Fig. 1:**
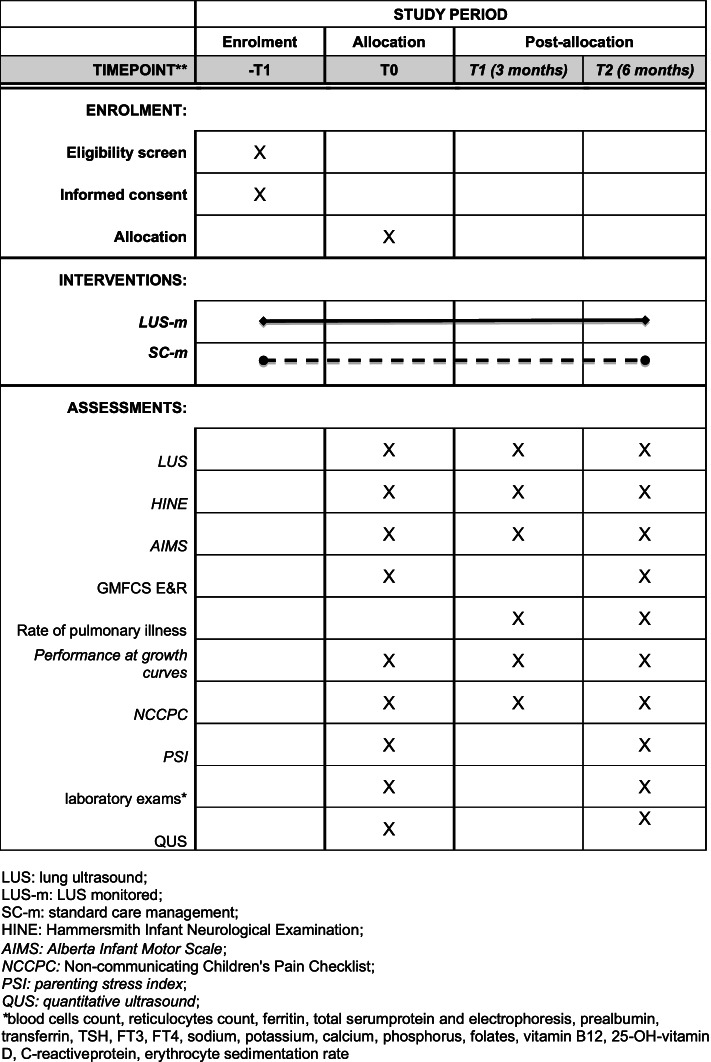
Schedule of study enrolment, interventions, and assessments

The presented protocol consists of a RCT, which compares LUS-monitored management of dysphagia-related aspiration versus standard care management in infants with neurological impairment. Study timeline is scheduled in Fig. 1.

The main aim of the presented trial is to explore the effectiveness of the use of LUS for monitoring pulmonary abnormalities related to aspiration during feeding in neurologically impaired infants, by comparing integrated LUS-monitored management of dysphagia to standard care in terms of medical/neurological/behavioral status, chronic pain/discomfort and stress for caregivers.

Specific hypotheses to be tested are:

1. Infants receiving LUS-monitored management will have lower rate of pulmonary illness and better growth curves, corresponding to less after-meal LUS abnormalities over time, compared to infants receiving standard care. They will also reduce the need for invasive diagnostic (VFSS/FEES) over time.
2. Infants receiving LUS-monitored management will have better results at blood sample and bone metabolism, lower pain indices and less stressful interaction with caregivers.

We also hypothesized that diagnostic benefits of LUS-monitored management will contribute to better neurological outcome.

Further aims also include the comparison between LUS and VFSS/FEES in the detection of feeding-related silent or apparent aspiration and to estimate aspiration entity by cut-off values for the diagnosis of clinically significant silent or overt aspiration, responsiveness of LUS monitoring to medical, postural or food consistencies adaptation, and their relationship with standardized clinical feeding evaluation and medical/behavioral/neurological measures. Moreover, we will explore the impact of brain abnormalities detected by brain MRI on LUS findings severity.

We will finally establish risk categories for unsafe swallowing disorders and potential clinical recommendations for early management.

### Participants

A minimum sample size of 126 subjects was determined (63 each group). Newborn and infants will be recruited by the child neurologist and the speech and language pathologist (SLP) from the Infant Neurology Section of academic hospital IRCCS Stella Maris Foundation in Pisa, Italy.

Subjects will be included in the study if: i) they have, or they are at risk for, cerebral palsy [15] or have abnormal muscular tone at any age between 0–6 years of life due disorders other than cerebral palsy; or ii) motor developmental delay assessed by a quantitative scale for infants and young children development (< 5 sd according to age); or iii) in absence of the previous clinical indices, if there is the suspicion of GERD or dysphagia based on clinical symptoms; iv) a brain MRI acquisition done before or programmed prior the end of the study period as part of their diagnostic procedure. Subjects will be excluded in presence of primary chronic lung diseases.

### Procedure

The medical staff of the recruiting center will refer eligible infants to researchers. Researchers will provide parents/guardians detailed information about the study procedures and seek informed consent. Assessments and procedures included in the study will be provided at IRCCS Stella Maris Foundation and Azienda Ospedaliero-Universitaria Pisana (AOUP), Pisa.

Also, the portable LUS machines that is used will allow the opportunity for home LUS evaluation to improve compliance of the patients to the experimental follow-up period.

### Study design

The trial is conceived as a double-blinded randomized parallel-designed controlled trial (Consort checklist, [24]), with block randomization (blocks of size 4), in one of 2 groups: 1) LUS-monitored (LUS-m) management; 2) Standard care management (SC-m).

Demographic data and motor, medical, neurodevelopmental and other associated measures, including brain MRI data, will be collected after participants’ parents/guardians have provided informed consent.

After baseline assessment (T0), both groups will undergo an experimental 6-months follow-up. When possible, in order to increase patients’ compliance to the protocol, experimental evaluation will be included in the clinical follow-up of the subject. In the first 3 months, participants will be evaluated a minimum of 1 time per month, in-hospital, for a total of 3 LUS-monitored meal evaluations, plus baseline (T0). Three months (T1) will be the primary endpoint. A further 6-months LUS-monitored meal and clinical assessment (T2) will be delivered. Primary and secondary outcome measures will be collected at T1 (primary outcome measures endpoint) and T2 (stability of outcome) (Fig. 1).

Study characteristics are detailed in Table 1.

**Table 1:** Summary of trial characteristics

Recruiting	Information
Primary registry and trial identifying number	ClinicalTrials.gov Identifier: NCT04253951
Date of registration in primary registry	05 February, 2020
Source(s) of monetary or material support	Italian Ministry of Health
Primary sponsor	Italian Ministry of Health
Contact for public queries	SF, MD, PHD simona.fiori@fsm.unipi.it
Contact for scientific queries	SF, MD, PHD simona.fiori@fsm.unipi.it IRCCS Fondazione Stella Maris, Pisa, Italy
Public title	LUNCH—Lung Ultrasound for early detection of silent and apparent aspiratioN in infants and young CHildren with cerebral palsy and other developmental disabilities: study protocol of a randomized controlled trial
Scientific title	LUNCH—Lung Ultrasound for early detection of silent and apparent aspiratioN in infants and young CHildren with cerebral palsy and other developmental disabilities: study protocol of a randomized controlled trial
Countries of recruitment	Italy
Health condition(s) or problem(s) studied	Aspiration in pediatric cerebral palsy and developmental disabilities
Health condition(s) or problem(s) studied	Active comparator: *Lung ultrasound* monitored management
	Standard care
Key inclusion and exclusion criteria	Ages eligible for study: 0–6 years Sexes eligible for study: both Accepts healthy volunteers: no
	Inclusion criteria: infant and children < 6 years; with, or at risk for, cerebral palsy or abnormal muscular tone due disorders other than cerebral palsy; suspicion of GERD or dysphagia based on clinical symptoms
	Exclusion criteria: primary chronic lung diseases
Study type	Interventional
	Allocation: randomized intervention model. Double blind (caregiver, outcomes assessor)
	Primary purpose: early diagnosis
Date of first enrolment	April 2021
Target sample size	150
Recruitment status	Recruiting

### Sample size determination

The required sample size was calculated according to one of the primary outcome measures, which is the difference in LUS scores at T1 between assignment groups, based on preliminary results (alpha = 0.05, power (1-ß err prob) = 80%, effect size f = 1.6) (unpublished data).

Estimating a conservative attrition rate of 15%, our sample size will include 150 participants over 30 months.

### Randomization/blinding

After enrolment, participants will be randomly assigned to LUS-monitored or standard care group by the use of block-generator software. Both the clinicians who will perform and score LUS, and the patients’ family will be blinded to randomization.

The child neurologist and the SLP will be informed of the result of LUS only in the LUS-m group and will include that result to impact on feeding care (postural- hygiene care adaptation, thickening fluids or drugs available for GERD). LUS results in the SC-m group will be available only at the time of data analyses.

### Adverse events

Any adverse event or unintended effect associated with the intervention will be reported and reviewed by researchers. In case of detection of LUS pulmonary abnormalities that will be considered a medical emergency which compromises a participant’s safety, unblinding will be permissible. The child neurologist will be informed of the unlikely result and will inform the family for appropriate therapy.

### LUS procedure/Equipment

The intervention is a LUS-monitored (before and after) feeding trial (the “after” evaluation will be within 15 min the end of the feeding procedure). A further LUS evaluation will be performed at a distance of 3 h, before the next meal to check for resolution of eventual after-meal abnormalities. All meals will be monitored by pulse-oximetry and video recorded.

**Fig. 2:**
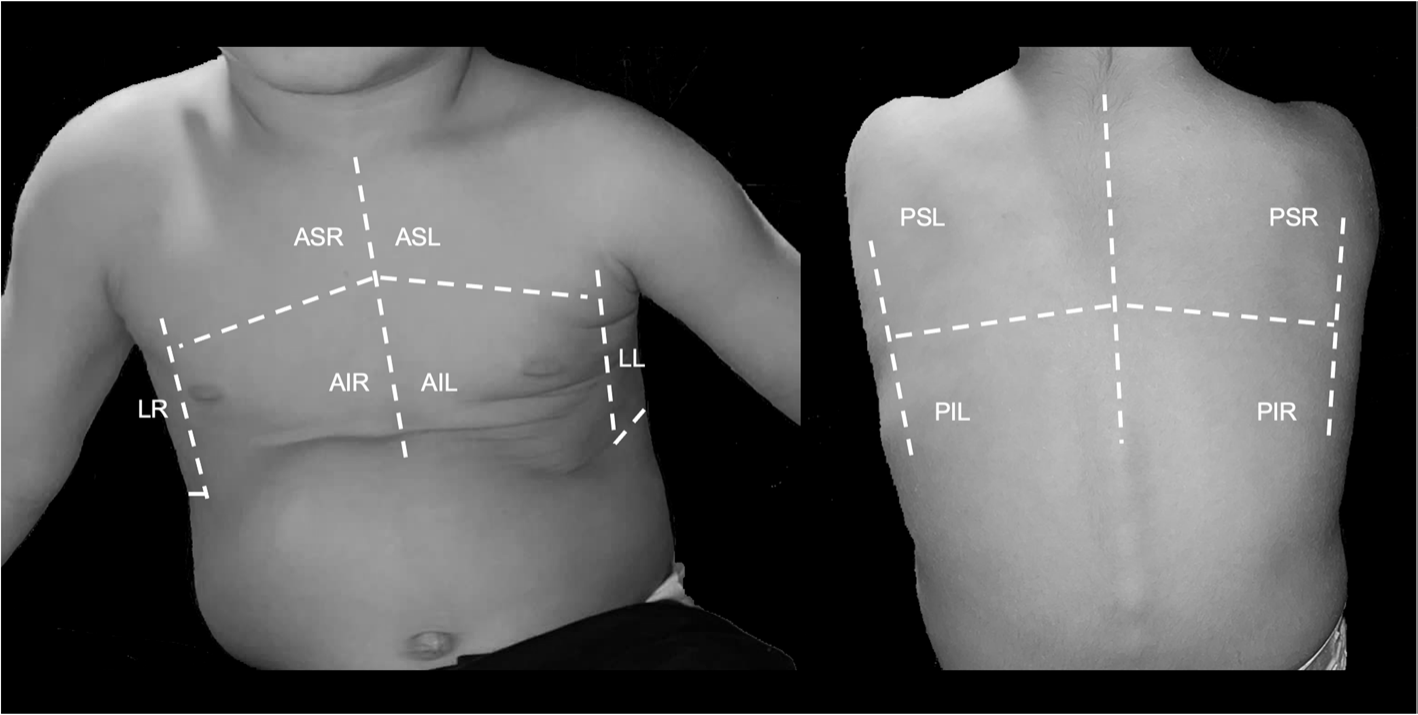
LUS scanning scheme. Ten scanning areas are identified. ASR: anterior superior right; AIR: anterior inferior right; ASL: anterior superior left; AIL: anterior inferior left; LR. Lateral Right; LL: Lateral left; PSR: Posterior Superior Right; PIR: Posterior Inferior Right; PSL: Posterior Superior Left; PIL: Posterior Inferior Left

Lung ultrasound exam will be performed based on a previously described scanning scheme in children [25], as indicated in Fig. 2. Commercially available portable machines with a linear probe (9–12 MHz) with specific setting for infants will be used (Lumify, Philips Medical Systems, Andover, MA, USA). Acquired images and movies will be saved in both groups but only available for final analyses in the SC-m group. In detail, the thorax will be divided into 3 major scanning areas (anterior, lateral, and posterior) divided by the parasternal, anterior axillary, and posterior axillary anatomical lines on both the right end the left hemithorax. A deaeration score will be used to classify each LUS exams, taking into account the following signs in each scanning area: the number of B-lines; the appearance of the pleural line; presence of C-lines; presence of small peripheral sub-pleural consolidations; presence of large consolidations; presence of pleural effusion. A deareation score will be calculated before and after the meal, so when the after-meal score will be different from pre-meal score (more than 10%), an intervention will be assigned to modify feeding performance and reported as medical, postural or food consistencies adaptation in the experimental group.

### Assessments

Infants will be assessed for outcomes according to the timeline. At T0, neurological information will be collected by using the Hammersmith Infant Neurological Examination (HINE) [26]; Alberta Infant Motor Scale (AIMS, [27]), GMFCS E&R [28]. The clinical measures used are standardized and validated for use across a wide age range.

Clinical primary outcomes will be rate of pulmonary illness (including pneumonia, wheezing, chronic cough, and apnea rate) and performance at growth curves (according to WHO growth standards, eventually age-corrected because of premature birth, or according to specific syndrome growth standards as recommended by ESPGHAN [9]. Between T0 and T2, parents will be asked to fill a detailed respiratory infection-diary for respiratory illness rate calculation (information collected at both T1 and T2). We will consider the exact, separate number of upper and lower airways infection and detect patients suffering from RRI (Recurrent Respiratory Infections), according to the most recent definition, i.e. i) 6 or more airways infection/year (at least 1 pneumonia, even severe), or ii) 2 pneumoniae even not severe but radiologically confirmed [29].

Primary outcome measure will include LUS score at T1. We assume that a better baseline detection of aspiration will result in better management with a reduction of pulmonary abnormalities detected by LUS at primary endpoint. Secondary outcomes collected at T1 will include chronic pain assessment (parents-report measures, behavioral measures including the Non-communicating Children’s Pain Checklist, and physiological measures) [30] and parents’ stress by a parent stress questionnaire (Parenting Stress Index PSI) [31]. At T0 and T2 laboratory exams will be performed to evaluate general and nutritional status (blood cells count, reticulocytes count, ferritin, total serum protein electrophoresis, prealbumin, transferrin, TSH, FT3, FT4, sodium, potassium, calcium, phosphorus, folates, vitamin B12, 25-OH-vitamin D, C-reactive protein, erythrocyte sedimentation rate, Insulin-like Growth Factor I) and quantitative ultrasound (QUS) also, in order to quantify bone mineralization.

VFSS and FEES data will be collected when performed according to multidisciplinary clinical indications, compared in frequency of execution between groups, and their results will be compared to LUS findings.

### Statistical analysis

Statistical analyses will be performed by using a statistical software (Statistical Package for Social Sciences, SPSS). Descriptive statistics and baseline between group differences will be explored. Statistical significance will be considered at *p* < 0.05. Post-hoc adjustment will be applied for multiple comparisons. Changes in LUS-score, growth and pulmonary illness rates at the primary endpoint (T1) will be calculated to assess the short-term effects of LUS-management versus standard care. Secondary outcome measures will be calculated at T1 and T2. Covariates such as type of neurological impairment, type/severity of brain lesion and age, will be included in the analyses. Multivariate statistics will be finally performed. Data imputation will be considered according to characteristics and relationship of missing variables.

### Ethics

The Paediatric Ethics Section of Tuscany Regional Ethics Committee on clinical trials (Italy) approved the presented study (study opinion registration number: 107/2019). Written consent will be obtained from parents of eligible infants, after being adequately informed about the trial by the Principal Investigator or the collaborators that have signed the ethical committee protocol. Relevant protocol modifications will be promptly communicated to the abovementioned Ethical Committee for approval revision.

According to recent recommendations on sensitive data management and patients’ privacy [32], an appropriate electronic password-protected access system for the correct de-identification/anonymization, collection, management, of patients’ data will be used. Participants’ identifying information will be stored separately with limited access.

## Discussion

We describe the rationale and protocol for a RCT that evaluate the effectiveness of LUS-monitoring for meal-related pulmonary abnormalities due to silent or apparent aspiration events and the advantages of its clinical use in the management of pulmonary-related consequences of dysphagia, due to silent or apparent aspiration, in infants with neurological impairment. Aspiration is indeed common in children with neurological impairment, leading to acute and chronic lung disease, malnutrition, further neurodevelopmental disturbances, chronic pain and stressful interactions with caregivers. No guidelines are available for monitoring and management, in particular for younger ages (up to 3 years), when x-Ray VFSS or FEES have prudent application. LUS may support diagnosis and management, reducing times, avoiding expensive and unsafe repetition of X-Ray examinations in a vulnerable population. LUS may advice short-term and long-term risk for apparent and silent aspiration, resulting in a tailored and patient-centered management. This repeatable, cost-effective, safe LUS-monitored feeding procedure might represent a reliable and promising measure to monitor postural, habilitative, pharmacological treatment effectiveness and to improve the effectiveness of the multidimensional assessment and treatment of chronic discomfort in such a population.

In sum, we expect LUS to allow earlier, faster, more complete monitoring and diagnosis in order to systematically prevent complications, but also contributing to a deeper knowledge of physiopathological mechanisms underlying pulmonary diseases in this vulnerable population and establish risk category for prognosis. LUS may optimize and rationalize resources, by reducing expensive and dangerous x-Ray examinations or more invasive techniques. These results might be also translated to low-resources countries and settings.

## Data Availability

The datasets generated and/or analysed during the current study are not yet publicly available as the recruitment is ongoing, but they can be available from the corresponding author on reasonable request. The Principal Investigators and the formal research collaborators will have access to the final trial dataset.

## Abbreviations

GERD: Gastroesophageal reflux
LUS: Lung Ultrasound
VFSS: Videofluoroscopic swallowing study
FEES: Fibre-opticendoscopic examination of swallowing
ESPGHAN: European Society of Gastroenterology, Hepatology, and Nutrition
RCT: Randomized Controlled Trial
MRI: Magnetic Resonance Imaging
